# Influence of menopause status on T-helper cell profiles in acute myocardial infarction

**DOI:** 10.17305/bb.2025.12354

**Published:** 2025-05-28

**Authors:** Fernanda Espinosa-Bautista, Varna Ramos-Rosillo, Yadira Vazquez-Panchos, Fernanda Bocanegra-Zamora, Héctor González-Pacheco, Mariana Patlán, Araceli Páez, Felipe Massó, Luis M Amezcua-Guerra

**Affiliations:** 1 Immunology Department, Instituto Nacional de Cardiología Ignacio Chávez, Mexico City, Mexico; 2 Coronary Care Unit, Instituto Nacional de Cardiología Ignacio Chávez, Mexico City, Mexico; 3 Basic Research Sub Directorate, Instituto Nacional de Cardiología Ignacio Chávez, Mexico City, Mexico; 4 UNAM/INC Translational Research Unit, Instituto Nacional de Cardiología Ignacio Chávez, Mexico City, Mexico; 5 Health Care Department, Universidad Autónoma Metropolitana-Xochimilco, Mexico City, Mexico

**Keywords:** Myocardial infarction, T-helper cells, menopause, inflammation

## Abstract

Estrogens modulate immune responses, particularly the activation and polarization of CD4^+^ T cells, which play key roles in cardiovascular homeostasis. This proof-of-concept study investigated the effect of menopausal status on the polarization of T-helper (Th) cells in women with acute myocardial infarction (AMI). A total of 41 female AMI patients were enrolled—seven premenopausal and 34 postmenopausal—and compared with a group of 17 male AMI patients. Flow cytometry was used to evaluate CD4^+^ T-cell subsets, including Th1 (T-bet^+^), Th2 (GATA3^+^), and Th17 (RORγt^+^) phenotypes. Serum levels of representative cytokines were also measured. Women exhibited higher numbers of circulating CD4^+^ T cells compared to men, with a marked shift toward the Th1 phenotype. Postmenopausal women demonstrated increased cardiovascular risk, as indicated by higher QRISK3 and GRACE scores, as well as elevated levels of C-reactive protein and cardiac troponin T compared to premenopausal women. However, menopausal status had minimal impact on Th cell polarization, as no significant differences were observed in the proportions of Th1, Th2, or Th17 subsets between premenopausal and postmenopausal women. Similarly, levels of interleukin (IL)-6, IL-1β, IL-10, tumor necrosis factor, and monocyte chemoattractant protein-1 were comparable between the two groups. This proof-of-concept study highlights sex-specific differences in immune responses and inflammatory profiles during AMI. Women exhibited a stronger polarization toward the Th1 phenotype, along with elevated markers of inflammation and myocardial injury. Notably, menopausal status did not significantly affect lymphocyte subpopulations or circulating cytokine levels.

## Introduction

Coronary artery disease (CAD) remains a leading global health concern, with acute myocardial infarction (AMI) representing it’s most severe manifestation [1]. Epidemiological data reveal significant sex-based differences in CAD prevalence, with premenopausal women exhibiting approximately half the risk compared to men [2]. This disparity diminishes after menopause, likely due to the loss of estrogen’s cardioprotective effects [2–4]. In women, regional fat accumulation—particularly in breast tissue—has been associated with increased cardiovascular risk, even before menopause, through the release of pro-inflammatory cytokines and activation of pro-apoptotic pathways [5]. These processes contribute to myocardial injury and impaired cardiac function. Chronic inflammation plays a central role in the pathogenesis of AMI and serves as a negative prognostic, diagnostic, and monitoring marker in CAD [6]. Myocardial ischemia and necrosis trigger a rapid inflammatory response that is essential for healing, with CD4+ T-helper (Th) cells playing a major role through their functional polarization [7]. In male patients with AMI, studies have consistently reported a dysregulated Th cell profile characterized by a Th1/Th2 imbalance and elevated Th17 responses [8, 9]. Estrogens modulate T-cell function through genomic mechanisms mediated by estrogen receptors (ERα and ERβ), which bind to estrogen response elements in the promoter regions of target genes [7]. These effects are complemented by non-genomic pathways involving mitogen-activated protein kinase (MAPK) and nuclear factor kappa B (NF-κB) signaling. Through these mechanisms, estrogens promote Th2-skewed responses by enhancing the production of interleukin (IL)-4 and IL-10, while suppressing Th1-associated cytokines such as interferon-gamma. Conversely, AMI has been associated with an increase in peripheral Th17 cells and Th17-related cytokines (IL-17, IL-6, and IL-23), along with reductions in regulatory T (Treg) cells and their associated cytokines, including IL-10 and transforming growth factor-beta [9]. Despite this knowledge, data on Th cell polarization in women with AMI remain limited, particularly regarding menopausal status and the immunomodulatory effects of estrogens. This proof-of-concept study aimed to investigate the polarization of Th1, Th2, and Th17 subsets in CD4+ T cells from women with AMI, with particular attention to differences between premenopausal and postmenopausal individuals.

## Materials and methods

### Study design

This single-center study enrolled 41 adult women admitted to the Coronary Care Unit for AMI, as defined by the Fourth Universal Definition of Myocardial Infarction [10], between January 2022 and April 2023. The hospital is a specialized university clinical center focused on cardiovascular diseases. AMI diagnosis was based on the presence of anginal symptoms along with evidence of myocardial injury, indicated by elevated cardiac troponin T (cTnT) or creatine kinase-MB (CK-MB) levels, with at least one measurement exceeding the 99th percentile upper reference limit. Exclusion criteria included ischemic symptoms lasting more than 72 h, pregnancy, active infection, malignancy, autoimmune or inflammatory disorders, blood dyscrasias, recent surgery, or end-stage kidney or heart disease. Eleven men were included as a comparison group and met the same inclusion and exclusion criteria, except for those specific to female sex. Natural menopause was defined as the absence of menstruation for at least 12 consecutive months, in the absence of medical or pathological causes [11]. Women receiving hormone replacement therapy (HRT) or hormonal contraceptives were excluded.

### Clinical assessments

Upon hospital admission, demographic and clinical data were collected, including the QRISK3 score—a validated algorithm for estimating the 10-year risk of developing CAD—and the GRACE score, a tool used to stratify the risk of in-hospital and post-discharge mortality [12, 13]. Coronary angiography was performed in the hemodynamics laboratory and interpreted by interventional cardiologists from our institution. Significant CAD was defined as greater than 50% luminal narrowing in a major coronary artery or the left main coronary artery. Patients were monitored daily until discharge, and the occurrence of major adverse cardiac events (MACE) was recorded. MACE included acute heart failure, pulmonary edema, recurrent myocardial infarction, cardiogenic shock, or death.

### Flow cytometry

Blood samples were collected within 60 min of admission to the Coronary Care Unit. Peripheral blood mononuclear cells (PBMCs) were isolated and analyzed using an FACSAria flow cytometer (BD Biosciences, San Jose, CA, USA). CD4+ cells were identified with antibodies specific to CD4 (PerCP; BioLegend, San Diego, CA, USA), T-bet (FITC; Th1 phenotype), GATA3 (PE; Th2 phenotype), and RORγt (APC; Th17 phenotype) (eBioscience, San Diego, CA, USA). Flow cytometry procedures and gating strategies were carried out as previously described [14]. All assays were performed by a single operator within 6 h of blood collection to preserve sample integrity, in accordance with best-practice guidelines [15]. Intra-assay variability ranged from 0.7% for GATA3-PE to 7.7% for RORγt-APC.

### Cytokine measurement

Serum levels of prototypical inflammatory (IL-6, IL-1β, tumor necrosis factor [TNF], and monocyte chemoattractant protein-1 [MCP-1/CCL2]) and anti-inflammatory (IL-10) cytokines were measured in duplicate using enzyme-linked immunosorbent assays (ELISAs; BioLegend). Serum samples were available only from female participants, as blood collected from male patients was anticoagulated with ethylenediaminetetraacetic acid (EDTA), which is incompatible with this assay.

**Table 1:** Main characteristics of the study participants

	**Participants grouped by sex**			**Women grouped by menopausal status**		
	**Men (*n* ═ 17)**	**Women (*n* ═ 41)**	*P*	**Pre (*n* ═ 7)**	**Post (*n* ═ 34)**	*P*
Age, in years	65 (45–81)	55 (24–68)	0.158	46 (24–52)	57 (47–68)	**<0.001**
Hypertension, *n*(%)	7 (41)	24 (58)	0.260	2 (28)	22 (64)	0.105
Diabetes, *n*(%)	8 (47)	24 (58)	0.563	3 (42)	21 (61)	0.421
Smoking, *n*(%)	11 (64)	21 (51)	0.397	2 (28)	19 (55)	0.237
Previous MI, *n*(%)	7 (41)	8 (19)	0.107	0	8 (23)	0.310
QRISK3 score, %	13.7 (3.0–50.4)	9.0 (0.1–28.2)	**0.037**	3.0 (0.1–13.1)	9.5 (1.2–28.2)	**0.018**
*Laboratory data at hospital admission*						
hsCRP, mg/L	2.6 (0.2–67.7)	5.3 (0.4–239.0)	**0.038**	5.0 (1.0-8. 2)	5.5 (0.4–239.0)	0.094
Glucose, mg/dL	144 (91–355)	161 (83–428)	0.500	132 (91–340)	165 (83–428)	0.392
Triglycerides, mg/dL	126 (63–304)	150 (54–449)	0.162	125 (68–413)	159 (54–449)	0.256
Total cholesterol, mg/dL	160 (95–276)	166 (81–252)	0.229	147 (81–218)	176 (93–252)	0.208
LDL-C, mg/dL	97 (34–222)	96 (29–177)	0.587	95 (43–146)	99 (29–177)	0.697
HDL-C, mg/dL	37 (27–49)	39 (27–84)	0.141	31 (27-49)	40 (27–84)	0.118
Hemoglobin, g/dL	16 (11–19)	14 (9–16)	**<0.001**	13 (11–15)	14 (9–16)	0.131
Leukocytes × 10^3^/mm^3^	10.5 (7.3–13.8)	11.6 (5.8–18.3)	0.239	11.7 (6.1–15.2)	11.2 (5.8–18.3)	0.469
Platelets × 10^3^/mm^3^	225 (113–308)	290 (110–702)	**<0.001**	249 (229–386)	295 (110–702)	0.183
Creatinine, mg/dL	1.0 (0.6–3.0)	0.7 (0.3–1.9)	**<0.001**	0.7 (0.5–1.0)	0.7 (0.3–1.9)	0.952
Albumin, g/dL	3.8 (3.1–4.9)	3.9 (2.7–4.6)	0.455	4.0 (3.5–4.49)	3.9 (2.7–4.6)	0.394
CK-MB, ng/mL	13 (2–300)	27 (0–300)	0.669	16 (1–124)	31 (0–300)	0.548
cTnT, pg/mL	196 (13–169 86)	1086 (67–324 30)	**0.025**	1018 (85–620 8)	1102 (67–324 30)	0.852
*Characteristics of myocardial infarction*						
Symptoms onset, *h*	4 (1-30)	7 (1-60)	0.113	6 (2-34)	7 (1-60)	0.979
NYHA ≥2, *n*(%)	6 (35)	14 (34)	>0.999	0	14 (41)	0.074
LVEF, %	40 (20–68)	50 (15–70)	**0.032**	52 (38–68)	50 (15–70)	0.214
STEMI, *n*(%)	17 (100)	30 (73)	**0.023**	6 (85)	24 (70)	0.651
GRACE score, points	118 (77–153 )	95 (64–178 )	**0.006**	76 (64–90)	100 (67–178 )	**0.004**
LAD artery occlusion, *n*(%)	12 (70)	24 (58)	0.553	3 (42)	21 (61)	0.421
Three-vessel disease, *n*(%)	3 (17)	11 (26)	0.523	1 (14)	10 (29)	0.651
*In-hospital drug therapies*						
Antiplatelets, *n*(%)	17 (100)	41 (100)	>0.999	7 (100)	34 (100)	>0.999
Heparin, *n*(%)	17 (100)	41 (100)	>0.999	7 (100)	34 (100)	>0.999
Statins, *n*(%)	17 (100)	39 (95)	>0.999	7 (100)	32 (94)	>0.999
RAAS inhibitors, *n*(%)	15 (88)	33 (80)	0.707	7 (100)	26 (76)	0.310
Thrombolytics, *n*(%)	8 (47)	23 (56)	0.574	3 (42)	20 (58)	0.678
Days of hospitalization	5 (1–9)	5 (1–44)	0.169	5 (4–11)	5 (1–44)	0.980

Data are expressed as the median (minimum–maximum range) unless otherwise specified. Significant *P* values are in bold. MI: Myocardial infarction; QRISK3: Cardiovascular disease risk algorithm 3; hsCRP: High-sensitivity C-reactive protein; LDL-C: Low-density lipoprotein cholesterol; HDL-C: High-density lipoprotein cholesterol; CK-MB: Creatine kinase-MB; cTnT: Cardiac troponin T; NYHA: New York Heart Association; LVEF: Left ventricular ejection fraction; STEMI: ST-elevation myocardial infarction; GRACE: Global Registry of Acute Coronary Events; LAD: Left anterior descending artery; RAAS: Renin-angiotensin-aldosterone system.

### Ethical statement

The study was approved by the local ethics committee (protocol codes 18-1089 and 21-1273) and adhered to the principles outlined in the Declaration of Helsinki as well as relevant local regulations. Written informed consent was obtained from all patients for the use of their clinical data and blood samples for research purposes. Medical interventions were administered solely at the discretion of the treating physicians, and the study did not influence patient treatment or clinical decisions.

### Statistical analysis

Data distribution was assessed using the Shapiro–Wilk test. Categorical variables were reported as percentages and analyzed with Fisher’s exact test. Continuous variables were expressed as medians with minimum–maximum ranges and compared using the Mann–Whitney *U* test. Correlation analyses were conducted using Spearman’s ρ coefficient, with 95% confidence intervals. All tests were two-tailed, and statistical significance was defined as *P* < 0.05. Statistical analyses were conducted using GraphPad Prism v9.3.1 (GraphPad Software, La Jolla, CA, USA).

## Results

The study included 41 women (median age: 55 years; range: 24–68), comprising seven premenopausal and 34 postmenopausal participants, along with a comparison group of 17 men (median age: 65 years; *P* ═ 0.158). Two women who had experienced amenorrhea for more than 12 but fewer than 36 months (borderline perimenopause) were classified as postmenopausal. Although demographic characteristics were comparable (Table 1), men exhibited higher QRISK3 scores (13.7% vs 9.0%; *P* ═ 0.037) and creatinine levels (1.0 vs 0.7 mg/dL; *P* < 0.001). In contrast, women had higher levels of cTnT (1086 vs 196 pg/mL; *P* ═ 0.025) and high-sensitivity C-reactive protein (hsCRP; 5.3 vs 2.6 mg/L; *P* ═ 0.038). Among women, postmenopausal participants were older (57 vs 46 years; *P* < 0.001) and had a higher cardiovascular risk (QRISK3: 9.5% vs 3.0%; *P* ═ 0.018) compared to premenopausal women. No significant differences were observed in AMI type (ST-elevation AMI: 85% vs 70%; *P* ═ 0.651), in-hospital therapies, or outcomes, including MACE (23.5% vs 42.8%; *P* ═ 0.360) and mortality (2.9% vs 0%; *P* > 0.999). However, postmenopausal women had a higher risk profile, as indicated by a greater predicted probability of in-hospital mortality (0.8% vs 0.3%) according to the GRACE score (100 vs 76 points; *P* ═ 0.004).

Flow cytometry results (Table 2) indicated that women had higher CD4^+^ T cell counts than men (3555 vs 2801 per 10,000 PBMCs; *P* ═ 0.003). Th1 polarization tended to be more pronounced in women (32.7% vs 28.8%), accompanied by a higher Th1/Th2 ratio (1.5 vs 1.1; *P* ═ 0.081). Th17 proportions were similar between sexes. Among women, CD4+ T cell counts were comparable between premenopausal and postmenopausal groups (3555 vs 3611 per 10,000 PBMCs; *P* ═ 0.622). Premenopausal women showed a slight predominance of Th17-polarized cells (55.0% vs 42.2%; *P* ═ 0.266), whereas postmenopausal women exhibited greater Th1 (33.9% vs 28.5%; *P* ═ 0.125) and Th2 (19.2% vs 16.8%; *P* ═ 0.799) polarization. However, the Th1/Th2 ratio remained similar between the groups (1.5 vs 1.5; *P* ═ 0.773). Stratified analyses showed that total CD4+ T cell counts were significantly lower in men compared to both premenopausal (*P* ═ 0.023) and postmenopausal (*P* ═ 0.007) women. No significant differences were observed in polarization profiles or in the Th1/Th2 ratio across these groups. No significant differences were found in serum levels of MCP-1/CCL2 (312 vs 234 pg/mL; *P* ═ 0.212), IL-6 (20 vs 38 pg/mL; *P* ═ 0.544), TNF (20 vs 7 pg/mL; *P* ═ 0.662), IL-1β (2.3 vs 5.1 pg/mL; *P* ═ 0.306), or IL-10 (3.9 vs 3.9 pg/mL; *P* ═ 0.409) between premenopausal and postmenopausal women. Additionally, cTnT levels did not significantly correlate with hsCRP (*ρ* ═ 0.18, CI: −0.14 to 0.47), IL-6 (*ρ* ═ −0.14, CI: −0.45 to 0.20), IL-1β (*ρ* ═ −0.16, CI: −0.47 to 0.17), TNF (*ρ* ═ 0.11, CI: −0.23 to 0.42), or IL-10 (*ρ* ═ 0.08, CI: −0.25 to 0.40).

**Table 2:** Color-flow cytometry assays and cytokine levels among study participants

	**Participants grouped by sex**			**Women grouped by menopausal status**		
	**Men (*n* ═ 17)**	**Women (*n* ═ 41)**	*P*	**Pre (*n* ═ 7)**	**Post (*n* ═ 34)**	*P*
*Flow cytometry assays*						
# of CD4^+^ cells/10,000 PBMC	2801 (485–4332)	3555 (1266–8497)	**0.003**	3555 (2219–4900)	3611 (1266–8497)	0.622
CD4^+^T-bet^+^/CD4^+^ cells, %	28.8 (14.9–49.0)	32.7 (10.0–55.9)	0.184	28.5 (20.2–35.3)	33.9 (10.0–55.9)	0.125
CD4^+^GATA3^+^/CD4^+^ cells, %	23.5 (4.3–69.5)	18.4 (6.2–54.3)	0.138	16.8 (9.6–54.3)	19.2 (6.2–38.0)	0.799
CD4^+^RORγt^+^/CD4^+^ cells, %	49.8 (14.1–64.9)	46.6 (20.0–82.5)	0.892	55.0 (24.3–65.1)	42.2 (20.0–82.5)	0.266
Th1/Th2 phenotype ratio	1.1 (0.2–6.9)	1.5 (0.3–7.2)	0.081	1.5 (0.3–3.6)	1.5 (0.5–7.2)	0.773
*Serum cytokine levels*						
MCP-1/CCL2, pg/mL	–	–		312 (177–500)	234 (45–500)	0.212
Interleukin-6, pg/mL	–	–		20 (7–160)	38 (7–360)	0.544
TNF, pg/mL	–	–		20 (7–126)	7 (7–130)	0.662
Interleukin-1β, pg/mL	–	–		2.3 (2.0–22.6)	5.1 (2.0–31.4)	0.306
Interleukin-10, pg/mL	–	–		3.9 (3.9–22.1)	3.9 (3.9–149.1)	0.409

Data are presented as the median (minimum-maximum values). Significant *P* value is in bold. PBMC: Peripheral blood mononuclear cell; TNF: Tumor necrosis factor; MCP: Monocyte chemoattractant protein-1; Th: T-helper.

## Discussion

This study aimed to investigate sex- and menopausal status-based differences in lymphocyte phenotypes among patients with AMI. Our findings revealed that women exhibited higher numbers of circulating CD4+ cells compared to men, with a distinct polarization toward the Th1 phenotype. Intriguingly, menopausal status appeared to exert no significant influence on CD4+ cell subpopulations or levels of circulating inflammatory mediators, suggesting that other factors may play a dominant role during the early phases of AMI. CD4+ cell polarization is heavily influenced by both inflammatory signals and the hormonal environment [16]. Estrogens are critical regulators of this plasticity: lower levels favor pro-inflammatory subsets (e.g., Th1 and Th17), while higher levels promote anti-inflammatory phenotypes (e.g., Th2 and Tregs). This adaptability is essential to the healing process following AMI, where pro-inflammatory subsets contribute to debris clearance, and anti-inflammatory subsets facilitate tissue repair and fibrosis [7]. Tregs are particularly important for controlling excessive inflammation and promoting cardiac healing, with implications for post-AMI outcomes and ventricular remodeling [17–18]. Evidence suggests that women, especially premenopausal women, may exhibit stronger Treg responses than men, potentially reflecting the immunomodulatory effects of estrogen and progesterone [16]. In our cohort, however, women displayed greater Th1 polarization and a higher Th1/Th2 ratio than men, aligning with previous findings that associate Th1-dominant responses with adverse cardiac outcomes [19]. Interestingly, we observed no significant differences in T cell polarization between premenopausal and postmenopausal women, although Tregs were not evaluated. This raises the possibility that the acute inflammatory milieu of AMI may override hormonal modulation or that similar clinical and angiographic characteristics of CAD across these groups may mask subtle hormonal effects. Another key observation is that women demonstrated higher levels of hsCRP and cTnT compared to men, despite having a lower baseline cardiovascular risk. This finding is consistent with previous studies showing elevated baseline hsCRP levels in women, particularly among certain populations, such as African Americans [20, 21]. Elevated hsCRP in women has been shown to predict cardiovascular disease independently of lipid levels and is strongly associated with increased in-hospital mortality during AMI [21–23]. Although hsCRP levels were similar between premenopausal and postmenopausal women in our study, postmenopausal women exhibited significantly higher cardiovascular risk, approaching levels observed in men. Regarding cTnT, while baseline concentrations are typically higher in men, cTnT levels are more predictive of cardiovascular events in women [24]. This discrepancy may relate to differences in cardiac mass, the prevalence of subclinical CAD, and the protective effects of estrogens, which influence atherosclerosis risk factors, thrombus formation, vasoreactivity, and vascular apoptosis [25, 26].

Our observation of an elevated cardiovascular risk profile in postmenopausal women aligns with prior epidemiological evidence suggesting a convergence in the incidence of CAD between sexes following menopause [2, 3]. Studies examining cardiovascular risk factors in premenopausal women have identified dyslipidemia, heavy smoking, hypertension, and diabetes as key contributors [27, 28]. These risk factors were prevalent in our study population and were notably more common among postmenopausal women. Angiographic studies have revealed distinct CAD presentation patterns based on menopausal status, with premenopausal women more frequently exhibiting single-vessel involvement, particularly in the left anterior descending artery [29, 30]. Our findings indicate that postmenopausal women exhibit a greater atherosclerotic burden and more severe CAD, underscoring the importance of including menopausal status in risk stratification and management strategies for women with CAD [31]. While total or near-total coronary occlusion is the most common angiographic finding in STEMI, it is less frequently observed in other forms of AMI. In our cohort, approximately one-quarter of patients did not present with STEMI, and several exhibited obstructive lesions involving less than 50% of the vascular lumen. Notably, two patients met the criteria for myocardial infarction with non-obstructive coronary arteries (MINOCA). Additionally, most patients received pharmacological reperfusion therapy prior to angiography, suggesting that some nonsignificant occlusions may reflect partial recanalization following fibrinolysis. Importantly, we observed no differences in leukocyte subpopulations between patients with vascular occlusions above or below the 50% threshold (data not shown). Although premature ovarian failure is associated with increased cardiovascular risk, HRT has not been shown to mitigate this risk, regardless of its timing or duration [32, 33]. Furthermore, data from the Women’s Health Initiative indicate a modest but statistically significant increase in CAD risk among postmenopausal women undergoing long-term HRT [34]. In contrast, emerging evidence suggests that metformin may modulate the inflammation involved in CAD pathogenesis and improve clinical outcomes in AMI patients [35]. We acknowledge that the small sample size may have limited our ability to detect subtle differences in CD4+ T cell subpopulations by menopausal status. The low number of premenopausal women enrolled reflects the rarity of AMI in this demographic, which in turn highlights the unique value of the data collected. Another limitation is the availability of only a single-time-point cTnT measurement, precluding longitudinal analysis of cytokine-to-troponin correlations. Additionally, sex hormone levels and ovarian reserve markers (e.g., anti-Müllerian hormone or ultrasound-based assessments) were not obtained. While such measurements would have enhanced our mechanistic interpretation, they were precluded by the urgency of patient stabilization and the need to minimize blood sampling. Moreover, acute stress-related hormonal fluctuations may have further complicated interpretation of endocrine data in this setting. Lastly, body composition data (e.g., body mass index, waist-to-hip ratio, or breast fat metrics) were not collected, which may confound cytokine profiles, given the pro-inflammatory role of adiposity. These issues should be addressed in future prospectively designed studies.

## Conclusion

This proof-of-concept study highlights sex-specific differences in immune responses and inflammatory profiles during AMI. Women exhibited a pronounced polarization toward the Th1 phenotype, along with elevated levels of inflammatory and myocardial lysis markers. Interestingly, menopausal status did not appear to significantly influence lymphocyte subpopulations or cytokine levels; however, further studies are needed to confirm these preliminary findings.

## Data Availability

All data are available on reasonable request to the corresponding author.
